# Supplementary material for: Conditional chemoconnectomics (cCCTomics) as a strategy for efficient and conditional targeting of chemical transmission
Source: eLife. 2024 Apr 30;12:RP91927. doi: 10.7554/eLife.91927 (PMC11060718; doi:10.7554/eLife.91927)
Supplement: Figure 3—source data 3. [file elife-91927-fig3-data3.zip › Figure 3 source data3 part 2.zip!/Figure 3-Source data 3-part2/1053-1-CRISPResso_Report_mpeqg9da.zip!/CRISPResso_on_1053_3/9.Alleles_frequency_table_around_sgRNA_ggagcggaatctacaaaacg.pdf]

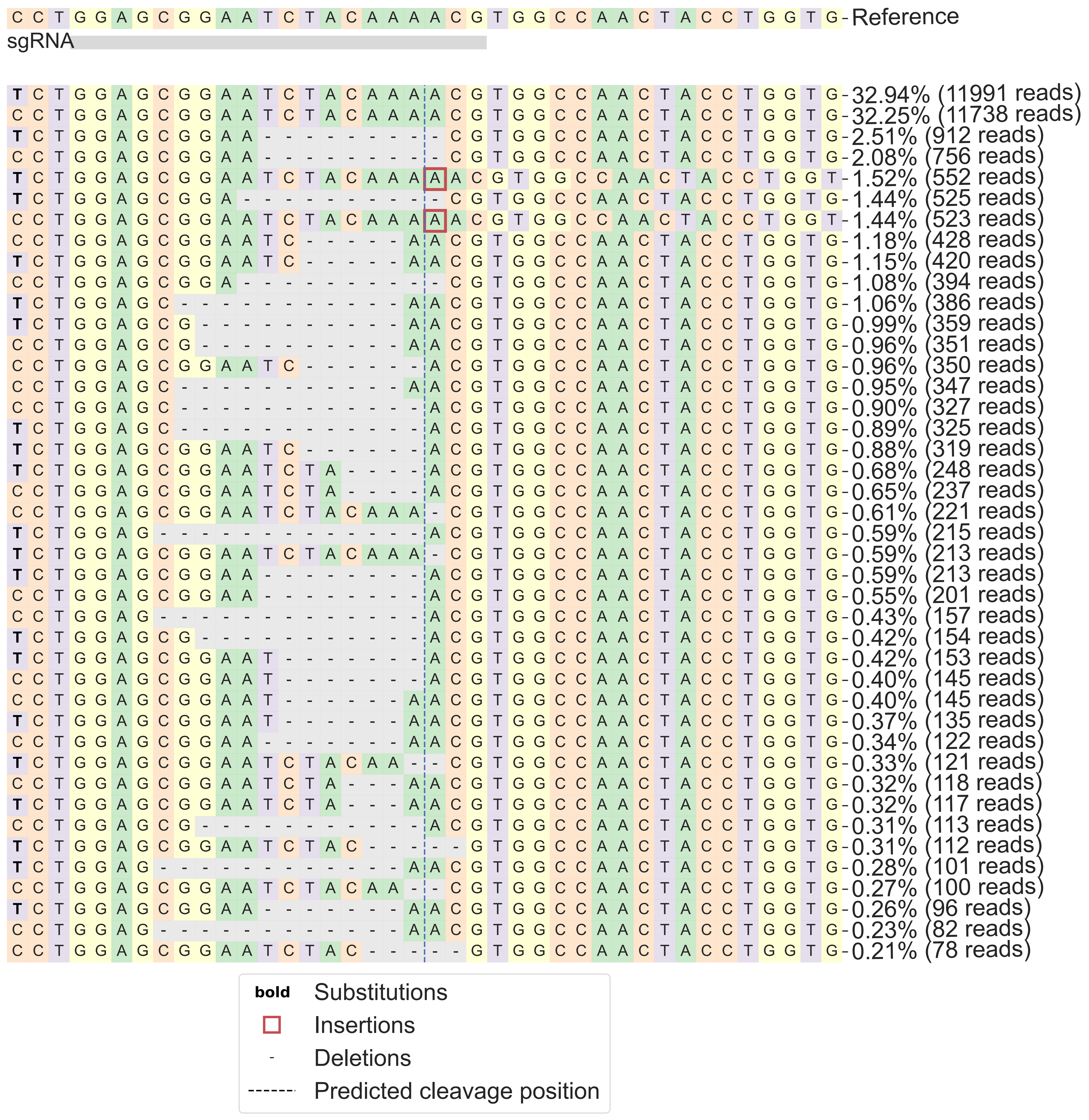
CCTGGAGCGGAATCTACAAAACGTGGCCAACTACCTGGTG - Reference
sgRNA
32.94% (11991 reads)
32.25% (11738 reads)
2.51% (912 reads)
2.08% (756 reads)
1.52% (552 reads)
1.44% (525 reads)
1.44% (523 reads)
1.18% (428 reads)
1.15% (420 reads)
1.08% (394 reads)
1.06% (386 reads)
0.99% (359 reads)
0.96% (351 reads)
0.96% (350 reads)
0.95% (347 reads)
0.90% (327 reads)
0.89% (325 reads)
0.88% (319 reads)
0.68% (248 reads)
0.65% (237 reads)
0.61% (221 reads)
0.59% (215 reads)
0.59% (213 reads)
0.59% (213 reads)
0.55% (201 reads)
0.43% (157 reads)
0.42% (154 reads)
0.42% (153 reads)
0.40% (145 reads)
0.40% (145 reads)
0.37% (135 reads)
0.34% (122 reads)
0.33% (121 reads)
0.32% (118 reads)
0.32% (117 reads)
0.31% (113 reads)
0.31% (112 reads)
0.28% (101 reads)
0.27% (100 reads)
0.26% (96 reads)
0.23% (82 reads)
0.21% (78 reads)
bold Substitutions
Insertions
Deletions
Predicted cleavage position
